# A Comparison between Passive airDNA Collection and Extraction Methods on the Recovered Terrestrial Vertebrate Assemblage

**DOI:** 10.1002/ece3.74135

**Published:** 2026-08-12

**Authors:** Jarred Moreno, Nicola Jackson, Celine H. Frere

**Affiliations:** ^1^ School of the Environment, University of Queensland St Lucia Queensland Australia

**Keywords:** airDNA, biomonitoring, DNA extraction, environmental DNA, metabarcoding

## Abstract

Over the past half‐decade, the collection of airborne environmental DNA (airDNA) has emerged as a novel tool for the biomonitoring of terrestrial vertebrates. Similar to other eDNA collection substrates (e.g., soil and water), the filtering of airDNA, while promising, requires rigorous methodological testing before widespread implementation. Two aspects of eDNA filtration that influence the recovered species assemblage are collection and extraction methods. Here, we assess the comparative performance of three collection matrices: cloth filters, paraffin gauze and PCR plate seals, alongside four readily available extraction kits: Qiagen DNeasy Blood & Tissue, Qiagen DNeasy PowerSoil Pro, MagMAX Total Nucleic Isolation and Quick‐DNA Miniprep Plus extraction kits. We deployed these passive airDNA filters in a riparian urban parkland within the Tarradarrapin Creek Wetlands in south‐east Queensland, Australia. Through the use of both a universal mammalian and universal vertebrate primer, we demonstrate the combination of cloth filters and the Blood & Tissue extraction kit records both the highest number of overall and informative terrestrial operational taxonomic units (OTUs) as well as a diverse and non‐variable species assemblage between replicates. Here, we show that optimising airDNA methodologies, and particularly using cloth filters with the Blood and Tissue extraction kit, allowed us to enhance the performance of this novel biomonitoring technique, a crucial step toward supporting its uptake alongside traditional monitoring methods (e.g., camera traps, bioacoustics or aquatic eDNA).

## Graphical abstract

Filtering airDNA, as with other biomonitoring tools, requires rigorous methodological testing before widespread implementation. Here, we assess the comparative performance of three collection matrices: medical gauze, paraffin gauze and PCR plate seals, alongside four readily available extraction kits: Qiagen DNeasy Blood & Tissue, Qiagen PowerSoil Pro, MagMAX Total Nucleic Isolation and Quick‐DNA Miniprep plus extraction kits. Through the use of both a universal mammalian and universal vertebrate primer, we demonstrate the combination of medical gauze and the Blood & Tissue extraction kit records both the highest number of overall and informative terrestrial operational taxonomic units (OTUs) as well as a diverse and non‐variable species assemblage between replicates.

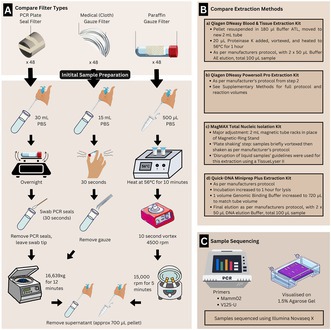

## 1 Introduction

Accurate biodiversity estimates are key to improving conservation planning (Bull et al. 2014). Methods for recording biodiversity are therefore many [e.g., camera traps (Wearn and Glover‐Kapfer 2019), live trapping (Thomas et al. 2020), bioacoustic arrays (Parsons et al. 2022) and detection dogs (Cristescu et al. 2015)], with airborne environmental DNA (airDNA) emerging as a new and promising tool for terrestrial species monitoring (Lynggaard et al. 2022). However, whilst seminal papers have demonstrated promising results (Clare et al. 2022; Clare et al. 2021; Frere et al. 2024; Garrett et al. 2023; Johnson et al. 2023; Lynggaard et al. 2022; Lynggaard, Calvignac‐Spencer, et al. 2023; Lynggaard, Frøslev, et al. 2023), airDNA is yet to undergo rigorous testing and optimisation to determine best practices. For comparison, the validation and optimisation of established aquatic environmental DNA, eDNA, technology has highlighted the necessary refinement of three essential methodological components: (1) the optimisation of DNA extraction methods to maximise yield and quality, (2) DNA collection methods and their effectiveness within natural environments and (3) primer selection to accurately distinguish key species of interest (Takahashi et al. 2023). It is critical to understand the performance of airDNA technology across these components as they have been shown to significantly impact the detection probability of species (Johnson et al. 2019).

To date, one study has compared extraction methods, primers and collection methods (i.e., trap type) for airborne plant eDNA. Here, Johnson et al. (2019) found that the detection of plant airDNA was most influenced by the extraction kit and primers, while the collection methods themselves performed similarly. Specifically, Johnson et al. (2019) recorded a greater DNA yield when using the Qiagen DNeasy PowerPlant Pro kit. Further, they argued that the performance differences between these kits could be attributed to PCR inhibition affecting air sampling (Johnson et al. 2019). Alongside these results, other studies have shown that the choice of extraction kit also depends on the intended target eDNA. For example, bead‐beating extraction methods reduce the detection probability of aquatic animal eDNA (Deiner et al. 2015), but not that of freshwater fungal eDNA (Matsuoka et al. 2022).

In addition to DNA extraction methods, identifying the most effective ways to collect animal eDNA from air particles will directly impact the efficacy and hence uptake of airDNA technology. For example, Bodawatta et al. (2026) demonstrated that high flow, 12 V fan filters with larger surface areas recorded a higher species richness than passive filters. However, their one‐to‐one comparison (one active to one passive) of airDNA collection performance did not account for how differences in sampling design (increased number of passive airDNA filters) could improve the performance of passive airDNA collection. For instance, passive filters are (1) more cost‐effective, (2) lighter weight, (3) easy to transport and deploy and (4) do not rely on a power source; all of which make them more scalable. Moreover, there are also differences in the ways we can collect airDNA passively. To date, researchers have collected airDNA passively by using paraffin coated microscope slides to collect airDNA deposited onto a media (Quesada et al. 2018), taking dust samples from a dust trap (Johnson et al. 2023), swabbing leaves (Lynggaard, Calvignac‐Spencer, et al. 2023), microfibre rollers (Guthrie et al. 2023), using sterile cloths (Frere et al. 2024) or using sterile forensic tape (Forsberg et al. 2016). Understanding how these collection media may impact the performance of airDNA remains to be investigated.

Here, we consider the in‐field performance of three previously used passive airDNA collection methods (Forsberg et al. 2016; Frere et al. 2024; Quesada et al. 2018) and four commonly used DNA extraction kits with a combination of a universal mammalian and universal vertebrate primer. Targeting animal communities, we aim to determine an effective and reliable method for (1) the collection of airDNA, (2) the extraction of airDNA and (3) increasing the breadth of species using multiple primers.

## 2 Methods

**FIGURE 1:**
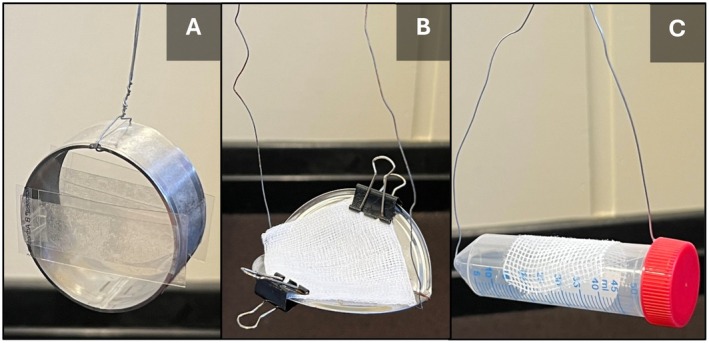
The three filter types used in this study, the PCR tape filter (A), the cloth filter (B) and the paraffin gauze filter (C).

We deployed three airDNA filter types (Figure 1; description in Filter Construction subheading), all designed to catch DNA both falling and being blown horizontally onto the filter. Namely, these filter types are (1) cloth (Frere et al. 2024), (2) paraffin gauze (Quesada et al. 2018) and (3) PCR plate seal filters (Forsberg et al. 2016). We deployed a total of 144 filters (*n* = 48 for each filter type) at Tarradarrapin Creek Wetlands (−27.501477, 153.227112) in the Redland Bay Catchment (SEQ, Australia) on the 10th of June 2024 for 2 days, collecting these on the 12th of June 2024. Sterile gloves and masks were worn for both the deployment and collection of the airDNA filters, with a different pair of gloves used to collect each individual filter. The filters were distributed among 13 trees within a 28 × 9 m area of the wetlands. The filters were deployed in triplicate (one of each filter type) on the same branch of a tree. Where possible, larger trees had multiple filter deployment sets (i.e., multiple branches with triplicates). The heights of each triplicate varied from approximately 20 cm to 1.5 m, as filter triplicates were placed opportunistically on tree branches. Upon collection, the filters were immediately frozen at −20°C until extraction. Samples were collected under the animal ethics number 2022/AE000765 from the University of Queensland.

### 2.1 Filter Construction

Three previously used airDNA filter types (Forsberg et al. 2016; Frere et al. 2024; Quesada et al. 2018), each with a different collection matrix, were deployed for comparison in this study. The first utilised adhesive PCR plate seals (four portions of 6 × 3 cm) (Forsberg et al. 2016) attached to a metal cylinder (Figure 1A), the second utilised 7.5 × 7.5 cm Leukoplast sterile compress cotton gauze, hereafter referred to as cloth, attached to a stainless‐steel ring (Figure 1B) (Frere et al. 2024), while the third utilised a 5 × 5 cm Cuticell Classic Paraffin Gauze Dressing (Quesada et al. 2018), hereafter referred to as paraffin, placed atop a 50 mL falcon tube (Figure 1C), see Supplementary Methods for further details. All filters were sterilised and packaged as per Supplementary Methods.

### 2.2 DNA Extraction

**FIGURE 2:**
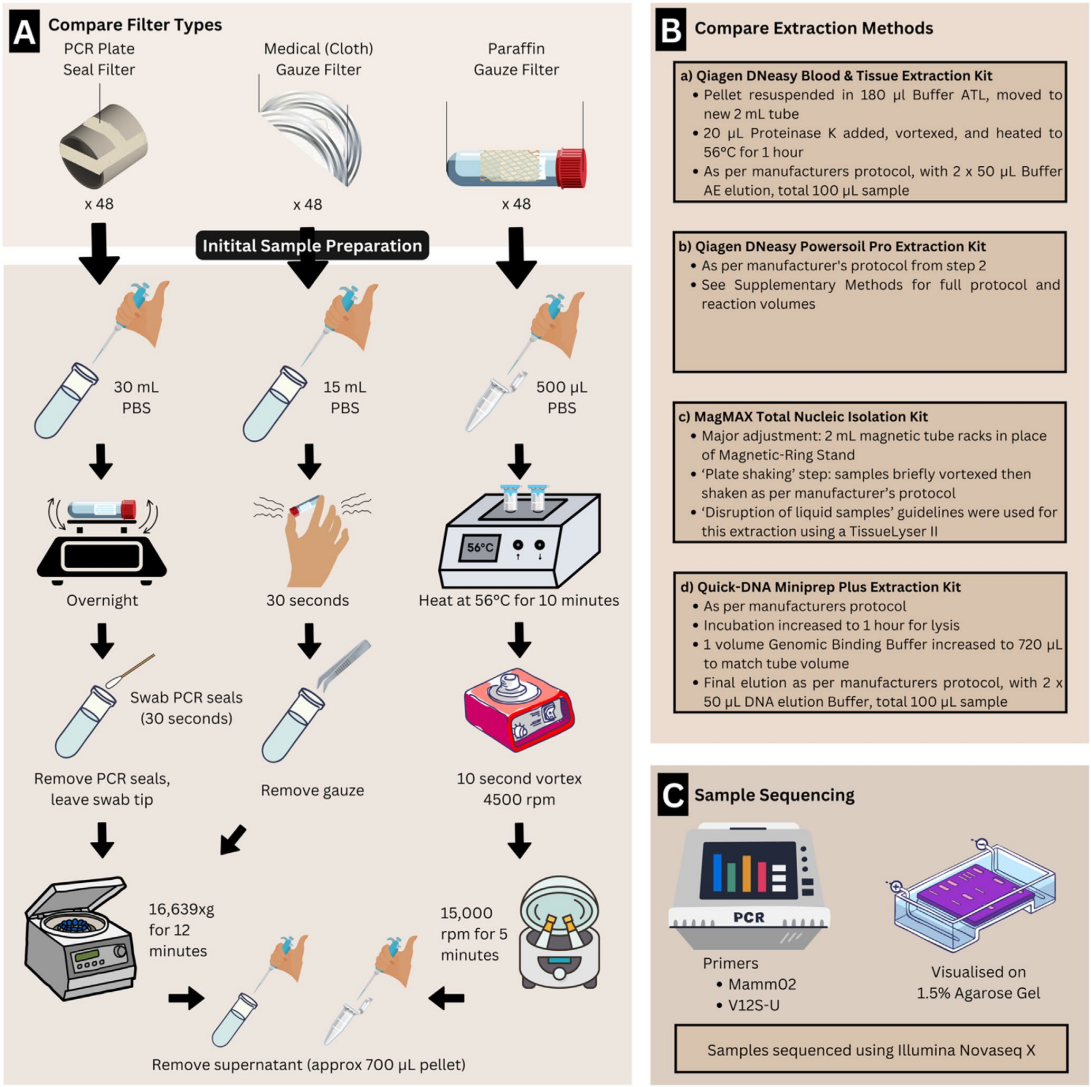
Graphical figure outlining the initial sample preparations for all three filtration methods, in order to standardise the samples to be processed through the four extraction kits. (A) General outline of the initial samples preparations for different filter types. (B) Outlines the changes to the manufacturers' protocols for each of the four extraction kits. (C) General overview of PCR, gel electrophoresis and sample sequencing.

A PCR clean room was used for the extraction and PCR amplification of all eDNA samples post collection, and gloves and masks were worn during both stages. Extraction and PCR amplification were also completed in sterile flow cabinets, as per the sterilisation procedure in Supplementary Methods, with the addition of an extraction blank in each set of 24 extractions. Due to the differences in collection substrates and differences in surface area, unique sample preparations were required for this standardisation of each method to be processed using each of the four extraction kits (Figure 2). Samples were chosen at random to be processed through each extraction kit to ensure no bias in location occurred during selection.

For each filter type, we compared four commonly used kits for DNA extraction: Qiagen DNeasy Blood & Tissue kit, Qiagen DNeasy PowerSoil Pro kit, MagMAX total Nucleic Acid Isolation Kit and Quick‐DNA Miniprep Plus (Zymo) kit (herein referred to as BT, PS, MM and ZY, respectively). These four kits all differ in their extraction methodology, and as such, modifications were made to each of the four extraction kits to standardise the process with the three filter types, see Supplementary Methods for more information. To summarise, the consistent changes across kits included the paraffin gauze being left in the tube during lysis, standardising the cell lysis to one hour, the final elution being kept at 2 × 50 μL elution with a 5‐min benchtop incubation prior to the first centrifuge, and finally, the kits with a bead‐beating method both used the same TissueLyser II protocol (Figure 2).

### 2.3 Metabarcoding

We used next generation sequencing (NGS) with two primers. First, the Mamm02 primer pair, which targets an average of 74 base pairs (bp) region of the 12S mitochondrial gene, Mamm02_FWD 5′‐CGAGAAGACCCTRTGGAGCT‐3′ (Taberlet et al. 2018) and Mamm02_RVS 5′‐CCGAGGTCRCCCCAACC‐3′ (Giguet‐Covex et al. 2014) alongside a human blocking primer MamP007_B_Hum1 5′‐GGAGCTTTAATTTATTAATGCAAACAGTACCC3‐3′ (Giguet‐Covex et al. 2014), designed to limit the amplification of human DNA within samples. For the initial PCR, we ran 20 μL reactions, consisting of 10 μL AmpliTaq 360 Master Mix (Applied Biosystems, Lithuania), 0.5 μL of a 10 μM forward primer, 0.5 μL of a 10 μM reverse primer, 2 μL of a 10 μM human blocker, 5 μL of nuclease‐free water, alongside 2 μL of template DNA. The Mamm02 thermocycling parameters were 95°C for 10 min, followed by 40 cycles of 95°C for 30 s, 57°C for 30 s and 72°C for 10 s and a final extension at 72°C for 7 min. Thermocycling parameters and cycles were optimised in‐house using gel electrophoresis to produce the brightest bands without non‐specific amplification. We found 40 cycles to be appropriate for this, as per Spitzer et al. (2025). The Mamm02 primer was selected due to its small amplicon size, its validation in previous use in eDNA studies (Mas‐Carrió et al. 2022), and as it was shown to amplify and distinguish local species, allowing for the best taxonomic classification. Second, the V12S‐U primer pair, which targets an average 208 bp region of the 12S mitochondrial gene, was selected, V12S‐U_FWD 5′‐GTGCCAGCNRCCGCGGTYANAC‐3′ and V12S‐U_RVS 5′‐ATAGTRGGGTATCTAATCCYAGT‐3′ (Wang et al. 2023). For the initial PCR, we also ran 20 μL reactions, however, without a human blocker, instead using an additional 2 μL of nuclease‐free water in its place. The V12S‐U thermocycling parameters were 95°C for 10 min, followed by 40 cycles of 95°C for 10 s, 53°C for 30 s and 72°C for 15 s, followed by a final extension at 72°C for 7 min. The same optimisation steps for the thermocycling parameters were applied for this primer, resulting again in 40 cycles being optimal, an extension of the optimisation steps by O'Connor et al. (2024). An additional sample of DNA isolated from a skin biopsy of an Indo‐Pacific bottlenose dolphin ( *Tursiops aduncus* ) was used as a positive control in each sequencing lane, as per the methods outlined in Frere et al. (2024). The dolphin control was collected under the animal ethics number 2022/AE000612 from the University of Queensland, as per Frere et al. (2024). All PCR plates were run in identical triplicates, and the PCR products from each were pooled for sequencing.

We visualised PCR products on 1.5% agarose gels and proceeded with sequencing all cloth and paraffin gauze samples with both primers, opting not to sequence any PCR filters due to large levels of non‐specific amplification and absence of amplification in extraction blanks. We also used Qubit 4 Fluorometry to measure the DNA concentration in each sample prior to sequencing, as per the Supplementary Methods. All subsequent samples were sent for two‐stage PCR to generate amplicons and for sequencing using the Illumina NovaSeq X platform at the Australian Genome Research Facility.

### 2.4 Bioinformatic Pipeline

All reads from both primer sets, Mamm02 and V12S‐U, were processed using the same pipeline. First, the forward and reverse reads were merged using usearch (version 11) (Edgar 2010) at a minimum match identity between the forward and reverse reads of 80%, and a maximum of 10 base pairs (bp) differences. Following merging, reads were trimmed, filtered for a minimum quality value of *q* = 20 and the relevant adapters were removed using cutadapt (version 4.2) (Martin 2011). The merged Mamm02 reads were trimmed to a maximum of 150 bp and a minimum of 50 bp, while the merged V12S‐U reads were trimmed to a maximum of 220 bp and a minimum of 130 bp. Reads were then collapsed into unique operational taxonomic units (OTUs) at a 95% similarity using BBMap (version 39.01) (Bushnell 2014) for both primer sets. Bowtie2 (version 2.4.5) (Langmead and Salzberg 2012) was then used to index and produce a mapping file for each unique OTU, after which BBMap was used to extract read coverage.

Following this, a local snapshot of the BLAST database (Altschul et al. 1990) created on 21/03/24 was used to perform a MegaBLAST, BLASTn and custom blast for each sample. We used this to produce the top 15 matches for each unique OTU, which allowed us to gain a better understanding of potential matches for OTUs with lower e‐values. The custom BLAST database was produced with a custom database of 12S, 16S, partial or complete genomes for 1431 species that are known to occur in Australia. These sequences were obtained through NCBI's GenBank (Clark et al. 2016). This enabled us to screen high‐quality matches quickly to collapse OTUs to species known to occur in the country. On occasion, this custom blast also enabled us to gain an understanding of potential matches for those OTUs with low percentage ID values (< 95%).

All OTUs in this project were manually collapsed. This was based on percentage ID, e‐value, number of bp differences to the reference sequence, and finally, similarity to the next closest matches. All species‐level assignments for both primer sets were made for those OTUs that had no more than 7 bp differences to the reference sequence. Further, a minimum of 95% identity was required between the OTU and reference sequence to be assigned at a species level, with other taxonomic ranks requiring lower percentage identities (i.e., genus = 90%–95%, family = 85%–90%, order = 80%–85%) In some cases, despite having higher than 95% similarity, only a genus‐level match could be assigned as references sequences were identical or had same number of bp differences to multiple species. This was frequently observed in samples matching to bird families, and as Mamm02 is designed to target mammals, there may not be enough diversity in this small region (~90 bp) to allow bird species to be assigned. Further, many domesticated species did not require a species‐level assignment (e.g., *Canis* sp., *Gallus* sp. and *Bos* sp.) as they are livestock and/or domestic pet species common throughout the Redland Bay area, and as such are collapsed into the domestic category. The remaining OTUs identified to mammals, birds or reptiles were collapsed into the informative species category. Finally, the dolphin positive control did show some contamination using the mammal primer, with a total read count of 7, and as such, all final assignments with a read count of 7 or lower were removed. No contamination was detected in the dolphin control with the vertebrate primer. Final filtered OTUs can be found in Table S1, while the fasta sequences for each OTU can be found in Table S2, and the NCBI accession numbers can be found in Table S3. These results are also summarised in Tables S4 and S5, which are used for the data analysis.

### 2.5 Analysis

The analysis for the OTU data statistics was performed in R version 4.5.1 (R Core Team 2024). The raw metabarcoding results were processed to calculate several per‐filter OTU richness metrics across four taxonomic subsets: total OTUs (all unique identifiable OTUs), named OTUs (those OTUs identified to genus or species), domestic OTUs (the subset of named OTUs that were identified to domesticated species), and informative species (the subset of named OTUs that were identified to non‐domesticated species). For each of these data subsets, these values were calculated for the combined primer dataset, as well as both primer datasets individually; see Table S6 for species counts for each group. The R package *readxl* (version 1.4.3) (Wickham and Bryan 2023) was used to import the Excel dataset, using the data in Table S4. All analyses were run for these three separate primer groups individually: combined, mammal (Mamm02) and vertebrate (V12S‐U).

From this, we applied generalised linear mixed models (GLMMs) using the glmmTMB package (1.1.14) (McGillycuddy et al. 2025) to test the relationship between the number of detected OTUs, filter types and extraction kits. Separate models were fitted for each primer group and each subset of OTUs, resulting in 12 total models. All models were run using an interaction term between filter type and extraction kit, to assess if there was an influence of either effect on the other, and the random effect of Tree_Number, as we wanted to assess if there was spatial variability in OTU richness across the site. Tree_Number was a unique identifier for each of the 13 trees that filters were placed on, with multiple filters on each tree. All four models used a Poisson distribution with a log link function based on initial observations of the spread of the data. The C|MM and P|MM combinations were excluded from all models using the informative OTU subset, as zero informative OTUs were recorded, making the combination uninformative and causing rank deficiency. Zero‐inflation was assessed for all models through the check_zeroinflation function in the performance package (0.16.0) (Lüdecke et al. 2021). Slight zero‐inflation was detected in models using the domestic OTU subset; zero‐inflated Poisson models produced worse model fit (higher AIC), and a large zero‐inflation component, thus retaining standard Poisson models. A final model was then produced for the total OTUs in all primer subsets. This model was run identically to Model 1, with the addition of a fixed effect, sample DNA concentration, to assess if DNA concentration influenced the number of OTUs in a sample.

All models were then visualised using the package *ggplot2* (version 3.5.1) (Wickham 2016), which allowed us to plot pairwise boxplots between filter type and extraction kit combinations. To visualise the pairwise difference between the interaction term in all models, we used the package *emmeans* (version 1.10.5) (Lenth 2024) with Tukey adjustment for multiple comparisons.

Species accumulation curves were produced for each filter type and extraction kit combination using the specaccum function in the vegan (version 2.6–8) (Oksanen et al. 2024) package, with method set to ‘random’ with 999 permutations. Curves were built using the unique taxa found in each of the 12 combinations of primer group and OTU subset. Mean and standard deviation for cumulative species richness at a maximum of 12 filters were visualised.

To understand the difference in species composition recorded between filter type and extraction kit combinations, we use the R package *vegan*. We applied the vegdist function to calculate the Jaccard distance to assess the differences in standardised species composition between filters. The differences in community composition between the combinations herein were tested using permutational multivariate analysis of variance (PERMANOVA) using the adonis2 function with 999 permutations. Following this, the pairwiseAdonis (version 0.4.1) (Martinez Arbizu 2020) package was used to understand the differences in standardised species composition between the unique combinations of extraction kits and filter types. We then combined this with the metaMDS function to perform non‐metric multidimensional scaling (NMDS) ordination for visualisation, with binary set to TRUE and seed 123 for replicability. Finally, we applied the betadisper function to assess the multivariate homogeneity of communities recorded between filters. First, we performed a PCoA with Cailliez correction, and scaled the eigenvalues for the first two axes by the square root to transform negative eigenvalues using the ape package (version 5.8‐1) (Paradis and Schliep 2018). Following this, the beta dispersion was calculated to investigate differences between the variance in species composition among replicates for the unique extraction kit and filter type combinations using Analysis of Variance (ANOVA). Non‐significant beta dispersion results indicate equal within‐group dispersion, supporting the interpretation that PERMANOVA results are true compositional differences rather than dispersion artefacts.

Following community analyses, the betapart (1.6.1) (Baselga et al. 2025) package was applied to partition the beta diversity into turnover and nested components. First, the overall multisite beta diversity was calculated using the beta.multi function with the Jaccard family, partitioning total dissimilarity (β_JAC_) into species replacement (β_JTU_) and nestedness resultant dissimilarity (β_JNE_). Pairwise dissimilarity between the unique combinations herein was calculated using the beta.pair function. To test significance between the filter type and extraction kit combinations on total beta diversity, turnover and nestedness, a PERMANOVA was run on each matrix using adonis2 with marginal effects (by = ‘margin’) and 9999 permutations. The total Jaccard distance matrix was square‐root transformed prior to analysis to improve the metric properties. Both the turnover and nestedness matrices were Lingoes corrected using the Lingoes function from the ape package to eliminate negative eigenvalues. Homogeneity of multivariate dispersion for each transformed matrix was assessed using the betadisper function, with the addition of the permutest function to assess significance by permutation with 9999 permutations. Some informative pairwise results showed numerical instability due to combinations with near‐zero distances, causing matrix singularity in the turnover and nestedness matrices.

## 3 Results

Our primary objective was to evaluate how filter type and extraction kit influence vertebrated species detections in airDNA samples, using two primer sets and partitioning the data into four subsets: (1) total (all OTUs regardless of taxonomic rank), (2) named (OTUs identified to a species or genus level), (3) domestic (OTUs taken from named subset that match to domesticated animal species) and (4) informative (OTUs taken from named subset that are not domesticated animal species). To assess methodological performance, we applied a suite of complementary metrics including species richness, community composition, beta diversity and species accumulation curves. For clarity, we present key patterns here, with full results and pairwise comparison provided in Tables S6–S18.

### 3.1 Species Richness

**FIGURE 3:**
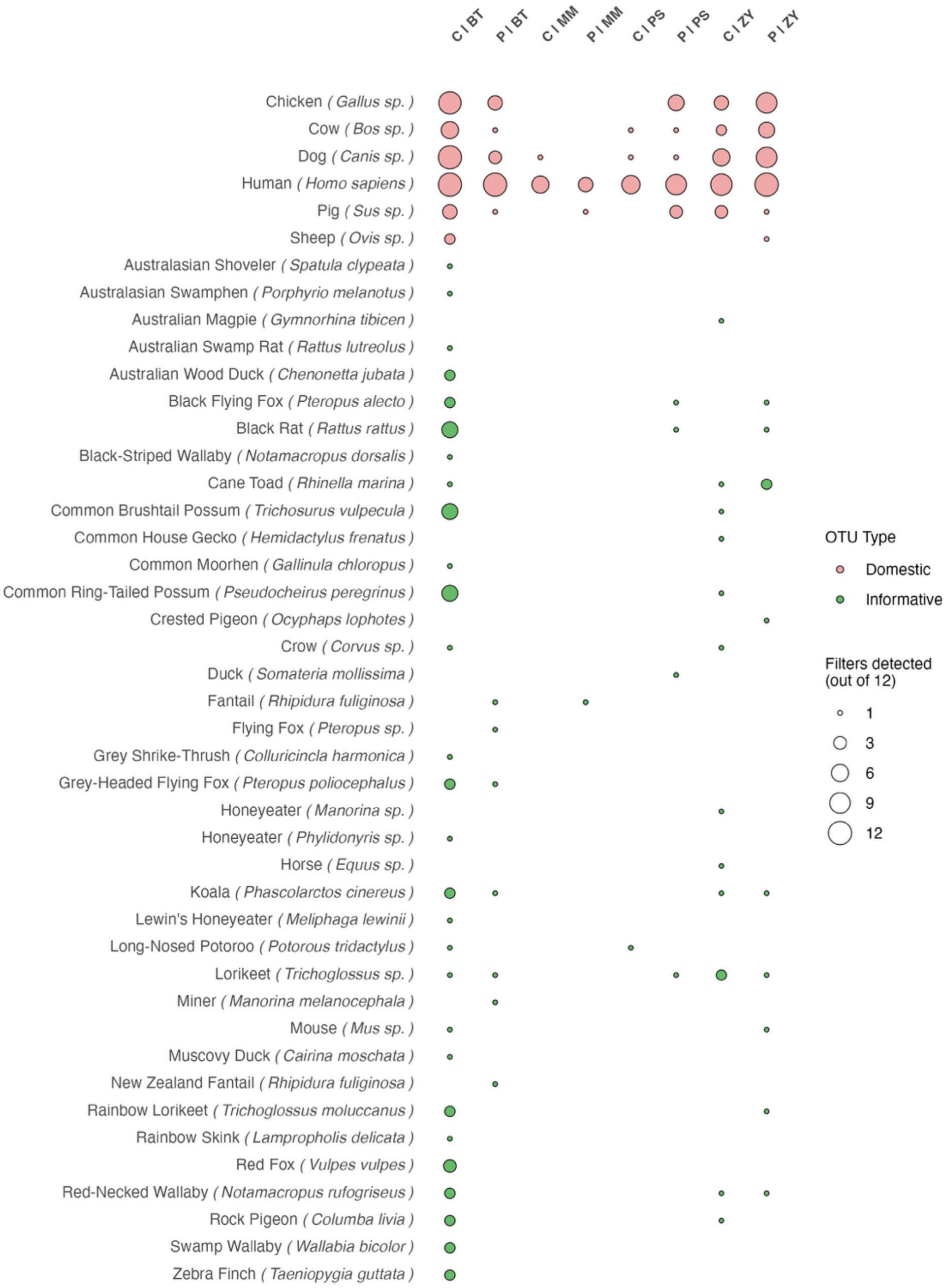
Bubble plot outlining the named OTUs (i.e., those identified to a genus or species level taxonomic assignment) found across the unique combinations of (C = Cloth, P = Paraffin) and extraction kit (BT = Blood & Tissue, MM = MagMax, PS = PowerSoil and ZY = Zymo). Bubble size is used to show the number of samples the species was identified in for each unique combination of filter type and extraction kit. Pink colouring of the bubble denotes domestic species, while green colouring denotes informative species.

Using both a mammalian and vertebrate primer, we were able to generate a total of 51 unique OTUs, 36 of which could be collapsed to a species level (Table S5). This included 17 OTUs unique to the mammalian primer dataset, 18 OTUs unique to the vertebrate primer dataset, and a further 16 OTUs being generated and shared between both primers (Table S5). A total of 15 OTUs could only be assigned to higher taxonomic classifications, including genus (*n* = 8), family (*n* = 6) and order (*n* = 1) (Table S5). From this, in the combined primer dataset, we record 51 total OTUs, 44 named OTUs, 6 domestic OTUs and 38 informative OTUs. The named OTUs (i.e., genus or species level assignments) can be visualised in Figure 3.

The total OTU richness differed significantly between the unique combinations of filter types and extraction kits. Firstly, all extraction kits, independent of filter type, recorded fewer OTUs than the Blood & Tissue kit (PowerSoil: *p* < 0.001, *z* = −7.431; MagMax: *p* < 0.001, *z* = −7.366; Zymo: *p* < 0.001, *z* = −5.006). Secondly, paraffin filters, independent of their paired extraction kit, reduced the number of OTUs detected relative to cloth filters for the total OTU (*p <* 0.001, *z* = −6.471), named OTU (*p* < 0.001, *z* = −5.954), domestic OTU (*p* < 0.002, *z* = −3.069) and informative OTU (*p* < 0.001, *z* = −5.034) subsets.

**FIGURE 4:**
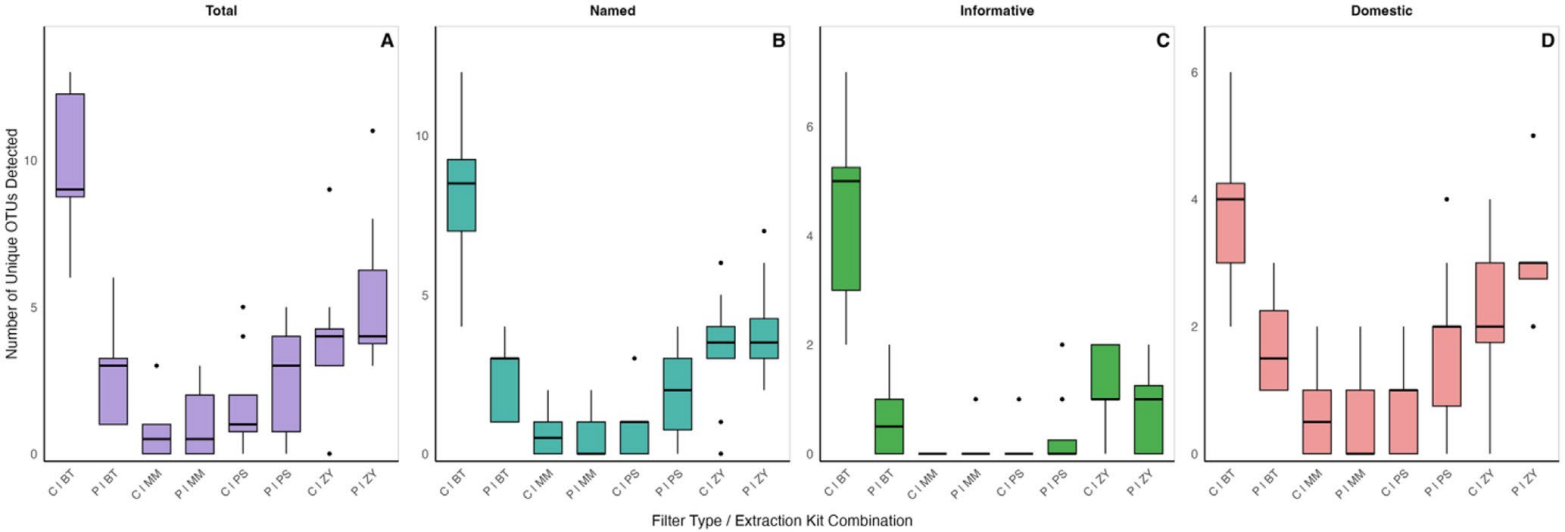
Boxplot demonstrating the median, interquartile range (IQR) and outliers for the number of unique identifiable OTUs using the combined dataset (Mamm02 and V12S‐U primer datasets) detected with each combination of filter type (C = Cloth, P = Paraffin) and extraction kit (BT = Blood & Tissue, MM = MagMax, PS = Powersoil and ZY = Zymo). The data is split into four panels demonstrating (A) the total OTU dataset, (B) the subset of total OTUs that are named to a genus or species level, (C) the subset of named OTUs that are identified to non‐domestic or informative species and (D) the subset of named OTUs that are identified to a domesticated species.

Following this, by interpreting filter types and extraction kits in combinations, we found that, for the combined vertebrate and mammal datasets, the C|BT combination recorded significantly higher species richness than all other combinations (Figure 4) for the total OTUs (all: *p* < 0.001), named OTUs (all: *p* < 0.001) and informative OTUs (all: *p* < 0.002, C|MM excluded). For the subset of domestic OTUs, C|ZY (estimate = 0.631, se = 0.258, *z* = 2.550, *p* = 0.175) and P|ZY (estimate = 0.239, se = 0.220, *z* = 1.088, *p* = 0.959, Table S9) recorded similar species richness.

Finally, the variance of the random effect of the tree ID was only important for the informative OTUs identified using the Mamm02 primer (variance = 0.037). Qubit DNA concentration was not a significant predictor of OTU richness (*p* = 0.191, *z* = 1.308, df = 79, Tables S7 and S8, Figure S1).

### 3.2 Community Composition

**FIGURE 5:**
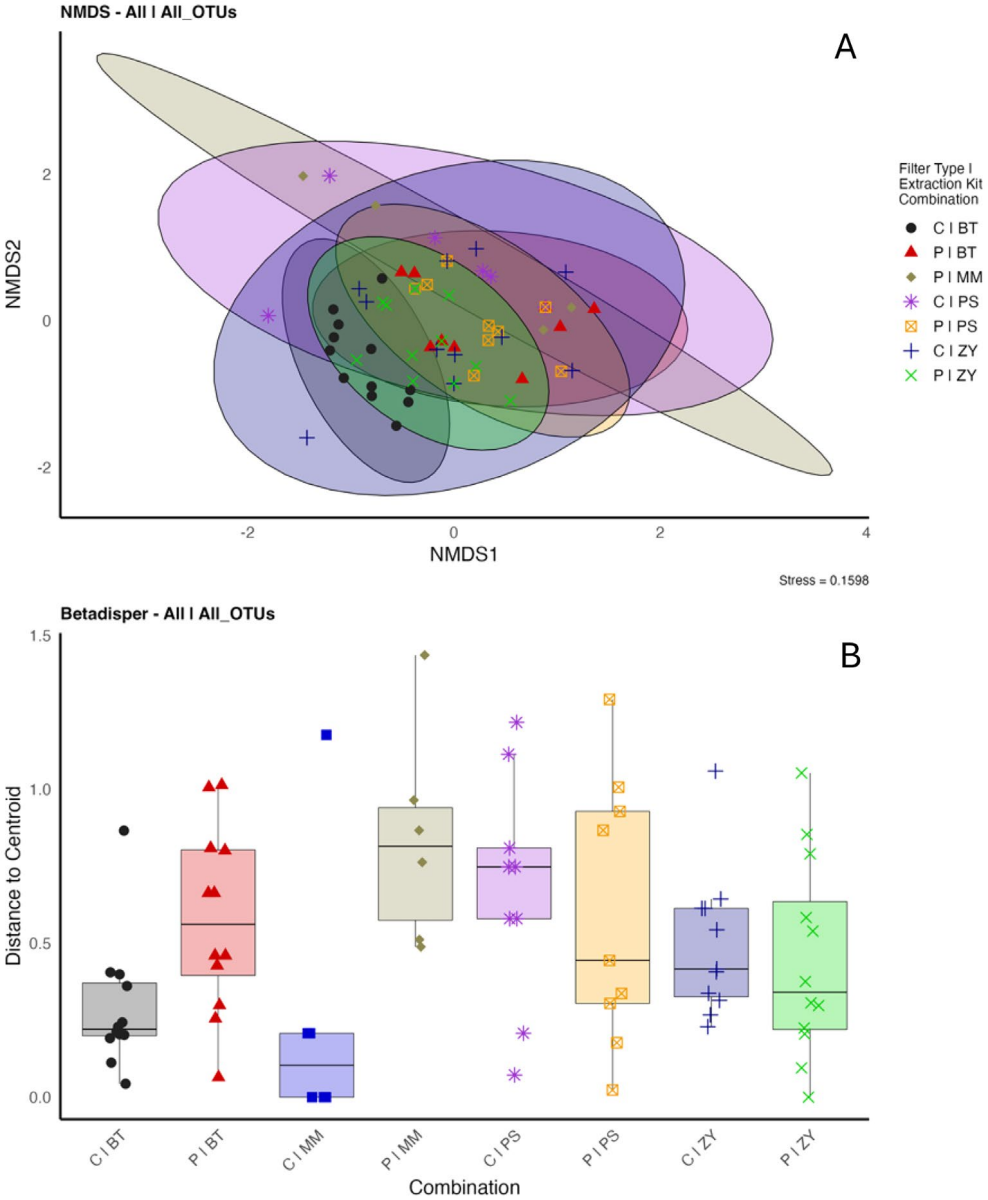
(A) Non‐metric Multidimensional Scaling (NMDS) ordination, representing the standardised species composition between the unique combinations of filter type (C = Cloth and P = Paraffin) and extraction kits (BT = Blood & Tissue, MM = MagMax, PS = PowerSoil and ZY = Zymo) for the total OTU dataset. NMDS ordination stress was 0.1598. Ellipses represent 95% confidence intervals (type = norm). (B) Box and whisker plot representing the spread or within‐combination variation of standardised species composition among replicates, demonstrated by distance to centroid, calculated from beta dispersion between replicates. This is represented for the total OTU dataset used in Panel A.

We observed similar trends between extraction kit and filter type combinations when examining community composition for both the combined and individual primer datasets. We found significant differences between combinations in favour of C|BT for the total OTUs (Figure 5), named OTUs and domestic OTUs (all *p* < 0.001, Table S10). For the informative OTUs however, we found non‐significant differences in community composition between C|BT and all other combinations for the combined (df = 6, *F* = 1.020, *R* ^*2*^ = 0.156, *p* = 0.376), mammal (df = 6, *F* = 0.931, *R* ^*2*^ = 0.177, *p* = 0.676) and vertebrate (df = 3, *F* = 1.008, *R* ^*2*^ = 0.137, *p* = 0.423) datasets.

Similarly to our species richness analyses, when we broke community composition metrics down to pairwise comparison between combinations of filter type and extraction kit, we again saw a trend toward the success of C|BT. Specifically, for the total, named and domestic OTUs, the C|BT combination recorded a unique community composition compared to all other combinations for the combined (all: *p* < 0.02, Table S11), and vertebrate (all: *p* < 0.002, excluding C|MM due to near‐zero vertebrate detections, Table S11) primer datasets. However, we found that in the mammal primer dataset, C|BT recorded a unique species community compared to most combinations (all: *p* < 0.005, Table S11), excluding C|ZY (total OTUs: df = 1, *F* = 1.429, *R* ^*2*^ = 0.064, *p* = 0.115; named OTUs: df = 1, *F* = 1.620, *R* ^*2*^ = 0.072, *p* = 0.078; domestic OTUs: df = 1, *F* = 2.287, *R* ^*2*^ = 0.075, *p* = 0.115) and P|ZY (total OTUs: df = 1, *F* = 1.155, *R* ^*2*^ = 0.050, *p* = 0.297; named OTUs: df = 1, *F* = 1.606, *R* ^*2*^ = 0.068, *p* = 0.092; domestic OTUs: df = 1, *F* = 0.766, *R* ^*2*^ = 0.034, *p* = 0.543).

Our differences in community composition were supported by the fact that we found non‐significant differences between the within‐combination beta dispersion between filter type and extraction kit combinations for the named, informative and domestic OTUs (all: *p* > 0.05, see Table S12 for pairwise comparisons). The total OTU dataset does demonstrate some differences in the beta dispersion of replicate filters OTU counts between replicates within combinations for both the combined primer (df = 7, *F* = 2.633, *p* = 0.017, Figure 5) and vertebrate datasets (df = 7, *F* = 2.380, *p* = 0.034), but remains non‐significant for the mammal dataset (all: *p* > 0.05, Table S12). However, we find this significance is attributed to the near‐empty C|MM OTUs, inflating the dispersion values, not attributed to a genuine biological pattern. Therefore, we are confident that our differences in community composition between combinations of filter types and extraction kits are true biological signals.

### 3.3 Beta Diversity

To complement the community composition analyses, we also assessed differences in beta diversity among all of our combinations. Overall, we detected a diverse community composition in our non‐domestic subsets of OTUs (i.e., total, named and informative OTUs). For both the total and named OTU subsets, the unique combinations of filter type and extraction kit detected different species, rather than subsets of each other's species communities (total OTUs: *β*_JAC_ = 0.891, *β*_JTU_ = 0.670 [75.2%], *β*_JNE_ = 0.221 [24.8%]; named OTUs: *β*_JAC_ = 0.875, *β*_JTU_ = 0.633 [72.3%], *β*_JNE_ = 0.242 [27.7%]). The domestic subset, however, showed that each combination herein was detecting a subset of the domestic species captured by C|BT, with zero turnover (*β*_JAC_ = 0.429, *β*_JTU_ = 0.000 [0%], *β*_JNE_ = 0.429 [100%]). Interestingly, the P|ZY and C|BT combinations record an identical domestic community composition (Tables S13 and S14).

In addition to the descriptive diversity statistics, we then partitioned these diversity metrics into two components, demonstrating that community composition differences, excluding informative species, found between combinations are entirely driven by nestedness (*β*_SNE_, where combinations are detecting a subset of another combinations' community) rather than turnover (*β*_SIM_, where combinations are detecting different communities of species compared to each other), for the combined primer dataset (total OTUs: *F* = 7.783, *R* ^*2*^ = 0.441, *p* < 0.001; named OTUs: *F* = 11.088, *R* ^*2*^ = 0.540, *p* < 0.001; domestic OTUs: *F* = 6.564, *R* ^*2*^ = 0.410, *p* < 0.001), the mammal primer (total OTUs: *F* = 8.708, *R* ^*2*^ = 0.480, *p* < 0.001; named OTUs: *F* = 9.854, *R* ^*2*^ = 0.519, *p* < 0.001; domestic OTUs: *F* = 6.933, *R* ^*2*^ = 0.431, *p* < 0.001) and the vertebrate primer (total OTUs: *F* = 4.207, *R* ^*2*^ = 0.357, *p* < 0.001; named OTUs: *F* = 7.488, *R* ^*2*^ = 0.533, *p* < 0.001; domestic OTUs: *F* = 5.976, *R* ^*2*^ = 0.482, *p* < 0.001) datasets. This confirms that any of the extraction kit and filter type combinations are detecting subsets of the C|BT domestic community composition, rather than detecting different species from each other. We find the opposite relationship for the informative OTU data subset, which showed the highest turnover of any subset (*β*_JAC_ = 0.889, *β*_JTU_ = 0.742 [83.4%], *β*_JNE_ = 0.147 [16.6%]). This turnover is, however, statistically non‐significant across all primer groups (both: *p* > 0.05, Tables S15–S17), with the exception of a weak nestedness signal in the combined dataset (*F* = 23.172, *R* ^*2*^ = 0.808, *p* < 0.001) that disappears when primers are analysed individually. This indicates that informative species detections are stochastic and not structured by combination choice, with no combination consistently detecting a different or more complete set of informative species than any other.

### 3.4 Species Accumulation

**FIGURE 6:**
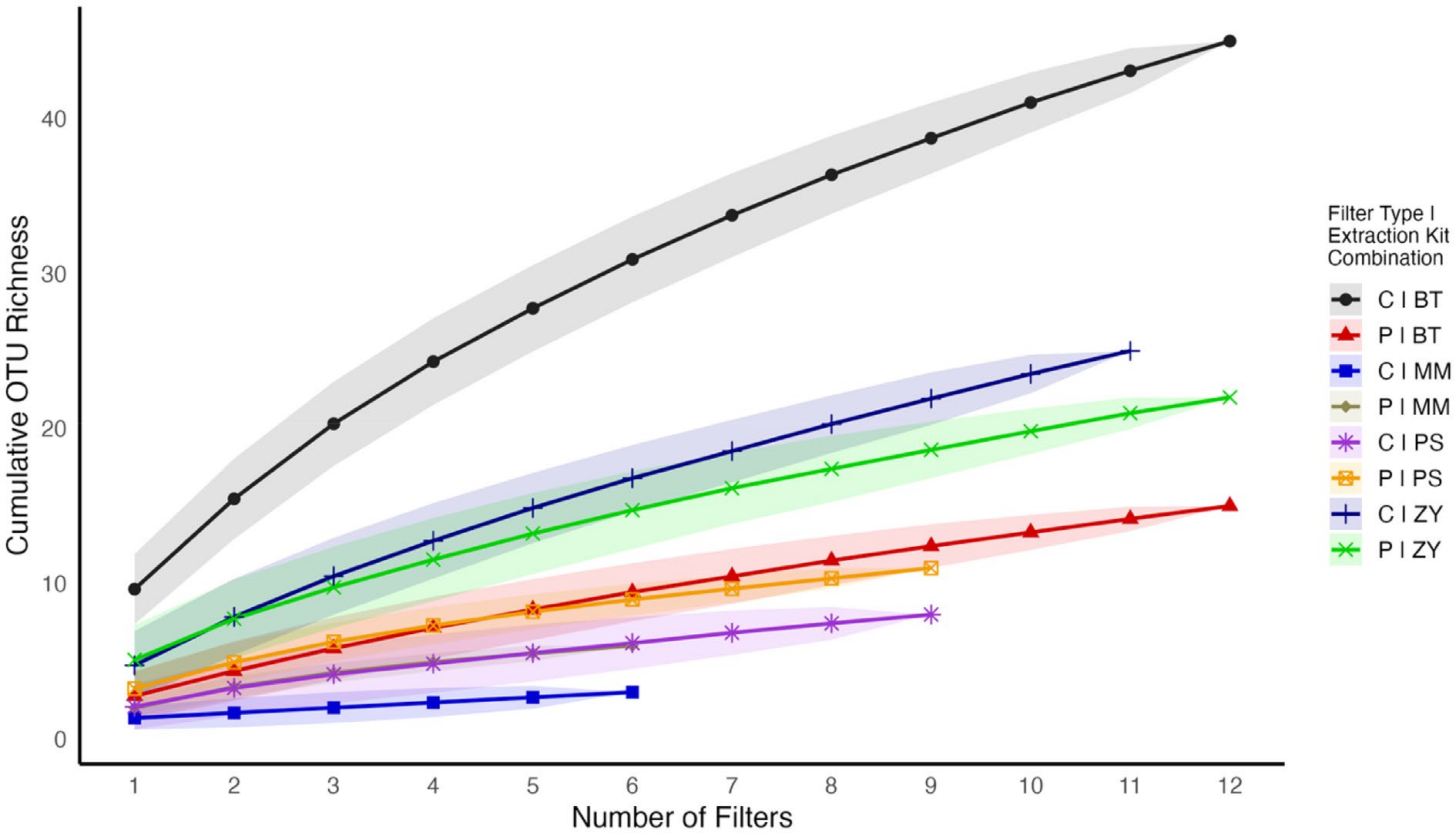
Species accumulation curves showing the cumulative OTU richness for the total OTU data subset, with coloured lines and symbols denoting the unique combinations of filter type (C = Cloth and P = Paraffin) and extraction kits (BT = Blood & Tissue, MM = MagMax, PS = PowerSoil and ZY = Zymo).

By using species accumulation curves, we see the same trend described by the other statistical tests herein. Specifically, the C|BT combination captures a more complete and diverse set of species than the others (Figure 6). Its accumulation curve reaches 45 OTUs with 12 samples, compared with 25 and 22 OTUs for the next best combinations (C|ZY and P|ZY). Even so, none of the curves level off, indicating that 12 filters are not enough to capture the full vertebrate diversity in this study (Table S18). Despite this, using 12 filters with C|BT still recovers about 88% of the total diversity detected across all methods (45 of 51 OTUs).

## 4 Discussion

In this study, we begin to build an understanding of how passive airDNA collection methods and DNA extraction kits may impact the detection of species through metabarcoding of airborne terrestrial vertebrate DNA. As with previous research comparing extraction kits and filter types for the extraction of airborne plant eDNA (Johnson et al. 2019), we were able to identify which combination of filter type and DNA extraction kit yielded (1) a consistently higher number of species and (2) best recovered low‐abundant informative species.

### 4.1 Filter Type and Extraction Kit Performance

Our results showed that the C|BT combination consistently outperformed the other combinations, recording both a higher number and greater diversity of species, that is, wider taxonomic breadth. While the C|BT performed better overall, we also found that this combination recorded a greater number of informative species, which is often the focus of airDNA research (Frere et al. 2024; Lynggaard, Frøslev, et al. 2023), without the loss of the domestic species. The ZY and BT extraction kits recorded comparable numbers of domestic OTUs. There is a trend throughout airDNA studies that domestic species are recorded ubiquitously in studies across multiple continents and consistently with high detection rates (Frere et al. 2024; Johnson et al. 2023; Lynggaard et al. 2022; Lynggaard, Frøslev, et al. 2023). This issue may be driven by the use of universal primers that are known to preferentially amplify highly abundant DNA (Piñol et al. 2015), which may lead to the masking of DNA sequences from rare species (Mas‐Carrió et al. 2022). Thus, when targeting rare or threatened species, the C|BT combination likely yields the best detection results.

The performance of C|BT became more obscure when looking at species communities compared to species richness. This is likely because although each combination records a subset of all the domestic OTUs detected by C|BT, we found that it was not the case for informative OTUs. Unlike domestic OTUs, we found that informative OTUs are spread more sporadically among all of our combinations, with most combinations detecting a unique informative OTU. Despite this, we still found that C|BT recorded overall more informative OTUs, though we note that some species are still not detected with this combination alone. This pattern may simply reflect an inherent limitation of airDNA for low‐abundance species, driven by the stochastic distribution of airborne particles (Kuparinen 2006). However, when we move beyond a single filter, we find that C|BT detects approximately 50% of our total recorded diversity with only five filters and more than 88% of our total recorded diversity with 12 filters. At five filters, the species richness was already higher than any other combination's total recorded richness with all 12 filters (Figure 6).

### 4.2 Refining Passive Sampling Methods

To encourage stakeholders' uptake of airDNA, minimising costs should be a primary objective. This is why we specifically chose to focus on passive airDNA optimisation, due to both its ease of deployment and cheaper construction cost (Frere et al. 2024; Jager et al. 2025; Newton et al. 2026). To do so, we chose to compare the efficacy of three pre‐validated passive airDNA methodologies (Forsberg et al. 2016; Frere et al. 2024; Quesada et al. 2018), each with varying degrees of filter viscosity. We hypothesised that the higher viscosity may result in greater DNA retention during collection. Our results, however, contrasted our initial prediction with our least viscous method, cloth, outperforming the other two filter types tested (paraffin gauze and PCR plate seal) for likely different reasons. First, cloth filters are larger (7.5 × 7.5 cm) than paraffin filters (5 × 5 cm), and larger filter surface areas have been previously documented to increase the recovery of airborne vertebrate DNA (Bodawatta et al. 2026). Second, cloth filters may have benefited from not being coated in a viscous medium after all, as the viscous medium of the paraffin gauze may have acted as a prohibitor during extraction and PCR, which Pogner et al. (2024) have shown in their recent work. Third, while the cloth filters outperform the other collection types irrespective of the extraction kit, the combination of C|BT also outperforms the other unique combinations. From this, we understand that while collection and extraction methods may drive community and richness differences individually, it may also be important to consider them in combination.

With a lack of a viscous matrix thought to play a role in the success of the cloth filter, we argue that cloth filters also outperform the PCR plate seal filters, despite not proceeding to sequencing. Our decision to exclude PCR plate seal filters from sequencing was based on a range of factors. Firstly, Collins et al. (2019) found that some universal primers using degenerate basepairs resulted in large amounts of wasted sequencing effort, with (a) low quality, (b) non‐target and (c) incorrect length sequences. When we visualised the post‐PCR samples by gel electrophoresis, the PCR plate seal filters amplified multiple different length amplicons within both primers, regardless of the extraction kit, which were absent for the other combinations. Sequencing these samples would likely result in the majority of reads being wasted on these factors, with many samples likely containing little to no usable sequences. Despite extensive optimisation of extraction methods and PCR conditions to minimise this non‐specific amplification, including (1) exclusion of a swab step, (2) longer and shorter lysing times and (3) addition of an inhibitor removal kit, we were unable to eliminate these off‐target products. Moreover, Shaffer et al. (2025) found significant effects of length bias toward shorter amplicons and GC‐bias, which can impact library preparation for NGS. As such, we made the decision not to sequence the PCR product, as it would have resulted in an expensive and uninformative sequencing effort if we were to proceed.

Beyond the selection of different filter media for comparing filter performance in eDNA studies, the methods used to process filters for DNA extraction may also play an important role. For instance, researchers could: (1) split/dissect each filter to be processed through each extraction kit (Johnson et al. 2019) or (2) use a whole filter for each extraction kit (Bodawatta et al. 2026). In this study, we chose to use the whole filter as we believed that splitting a filter would increase the risks of (1) causing mechanical damage to DNA, (2) losing DNA from the filter while handling it (Zaiko et al. 2022) and (3) introducing contamination between filters due to additional handling (Spens et al. 2017). We also argue that the increased labour and reagents required to split filters would raise costs and, as such, (1) reduce the number of biological replicates one could deploy and (2) limit the uptake of passive airDNA by stakeholders.

### 4.3 Future Directions

Our study is one key step in developing trust in this novel biomonitoring technology; however, further optimisation steps may help to increase the recovery of terrestrial vertebrates' DNA even further. The first consideration, for instance, would be to use molecular‐grade water in place of PBS during extraction, which has already been shown to decrease PCR inhibition (Sheth et al. 2022). Another area of optimisation would be to optimise a universal probe to calculate an accurate DNA quantification, rather than using a broader scale fluorometry, such as that used in this study. Bodawatta et al. (2026) have demonstrated the application of this technique, where combining a universal probe with their PCR, found strong support for higher DNA concentration being associated with more bird OTUs.

While the Blood & Tissue kit is widely used in airDNA studies focusing on terrestrial vertebrates (Clare et al. 2022; Frere et al. 2024; Garrett et al. 2023; Johnson et al. 2023; Lynggaard et al. 2022; Lynggaard, Frøslev, et al. 2023), our study has validated its advantages over other available extraction methods. Here we report that, when compared to the other combinations, the C | BT combination records (1) higher species richness (both per filter and overall) (Figure 4), (2) a higher number of informative, non‐domestic species (Figure 4), (3) a more complete species assemblage (Figure 6) and (4) high consistency in the species composition recorded among replicates (Figure 5). Whilst the verdict is still out on the comparative performance of passive and active airDNA sampling for terrestrial vertebrate species (Bodawatta et al. 2026; Jager et al. 2025; Newton et al. 2026), we do find that the refinement of passive airDNA methodologies greatly increased our species detections.

## Author Contributions

**Jarred Moreno:** conceptualization (equal), data curation (equal), formal analysis (equal), investigation (equal), methodology (equal), visualization (equal), writing – original draft (equal), writing – review and editing (equal). **Nicola Jackson:** conceptualization (equal), data curation (equal), formal analysis (equal), investigation (equal), methodology (equal), supervision (equal), visualization (equal), writing – original draft (equal), writing – review and editing (equal). **Celine H. Frere:** conceptualization (equal), data curation (equal), formal analysis (equal), funding acquisition (equal), investigation (equal), methodology (equal), project administration (equal), supervision (equal), visualization (equal), writing – original draft (equal), writing – review and editing (equal).

## Funding

The authors have nothing to report.

## Ethics Statement

Ethics approval was obtained for this project, under animal ethics number 2022/AE000765 from the University of Queensland.

## Conflicts of Interest

The authors declare no conflicts of interest.

## Supporting information


**Data S1:** Supplementary methods.
**Figure S1:** Dotplot showing the relationship between the total number of unique ASVs recorded on each sample and the QUBIT concentration for each sample, with the unique combination of filter type (circle = cloth, triangle = paraffin) and extraction kit (orange = Blood and Tissue, green = MagMAX, blue = PowerSoil or purple = Zymo) outlined.

**Table S1:** Metabarcoding results with collapsed OTUs, and BLAST taxonomic assignment information.
**Table S2:** Unique OTU sequences in fasta format represented in Tables S2 and S4.
**Table S3:** NCBI accession numbers for the species level assignments of OTUs in this dataset.
**Table S4:** The full table of results for each sample used for statistical analyses.
**Table S5:** Table of OTU assignments demonstrating the lowest taxonomic rank assignable for each OTU.
**Table S6:** OTU richness counts per filter sample recalculated from Table S1 using n_distinct (COMMON_NAME) per Sample. Total_OTUs = all unique species. Named = species identified to genus or species level, labelled informative or domestic. Informative = non‐domestic named species. Domestic = domestic named species only. Mamm02 = Mamm02 primer. Vert = V12S‐U primer. Used as response variables in glmmTMB richness models (S2–S3).
**Table S7:** Fixed effects from all glmmTMB species richness models (Poisson, log link, random effect: Tree_Number). Intercept = C|BT (Cloth|Blood and Tissue). Estimate = log‐scale coefficient. *P*, *p*‐value; SE, standard error; *z*, *z*‐statistic. Cloth|MagMAX excluded from Informative models (zero detections). Paraffin x MagMAX interaction dropped from Informative models (rank deficient). Primer = Combined, Mamm02, V12S‐U. Subset = total_OTUs, named, informative, domestic.
**Table S8:** Fixed effects from glmmTMB model testing QUBIT DNA concentration as a covariate of total OTU richness (Total_OTUs~QUBIT_ngmL + Filter_Type * Extraction_Kit + (1|Tree_Number), Poisson, log link). QUBIT_ngmL was not significant (*z* = 1.308, *p* = 0.191).
**Table S9:** All pairwise emmeans contrasts from glmmTMB richness models (emmeans package, Tukey adjustment). Estimates on the log scale. df = inf is expected for Wald‐type tests with glmmTMB. Primer = combined, Mamm02, V12S‐U. Subset = total_OTUs, named, informative, domestic.
**Table S10:** Global PERMANOVA results using adonis2() from vegan (Jaccard dissimilarity, binary = TRUE, 999 permutations). df, degrees of freedom; *F*, pseudo‐*F*; *P*, permutation *p*‐value. *R* ^2^, proportion of variance explained. Primer = Combined, Mamm02, V12S‐U. Subset = All_OTUs, named, informative, domestic.
**Table S11:** Full pairwise PERMANOVA for all combination pairs using pairwise.adonis2() from pairwiseAdonis (Jaccard, 999 permutations). df, degrees of freedom; *F*, pseudo‐*F*; *P*, permutation *p*‐value; *R* ^2^, variance explained. Primer = Combined, Mamm02, V12S‐U. Subset = All_OTUs, named, informative, domestic.
**Table S12:** Betadisper ANOVA results using betadisper() and anova() from vegan. PCoA with Cailliez correction applied prior to betadisper. Non‐significant (*p* > 0.05) = equal within‐group dispersion, supporting PERMANOVA. Primer = Combined, Mamm02, V12S‐U. Subset = All_OTUs, named, informative, domestic.
**Table S13:** Overall multisite beta diversity partitioning using beta.multi() from betapart (Jaccard index family), computed at the combination level. Beta_Total = total Jaccard. Beta_Turnover = replacement (Jtu). Beta_Nestedness = nestedness‐resultant (Jne). Turnover_Pct/Nestedness_Pct = percentage contribution of each component. Primer = Combined, Mamm02, V12S‐U. Subset = All_OTUs, named, informative, domestic.
**Table S14:** Full pairwise beta diversity partitioning between all combination pairs using beta. pair() from betapart (Jaccard), computed at the combination level. Turnover = Jtu. Nestedness = Jne. Total = Jac. Primer = Combined, Mamm02, V12S‐U. Subset = All_OTUs, named, informative, domestic.
**Table S15:** PERMANOVA testing differences in total (Jaccard), turnover (Jtu) and nestedness (Jne) beta diversity between combinations, following Baselga & Orme (2012). Sample level (96 filters), adonis2() by = margin, 9999 permutations. Jaccard sqrt‐transformed; JTU and JNE Lingoes‐corrected, Metric = component tested. *F*, pseudo‐*F*; *P*, permutation *p*‐value; *R* ^2^, variance explained. Primer = Combined, Mamm02, V12S‐U. Subset = All_OTUs, named, informative, domestic.
**Table S16:** Pairwise PERMANOVA on each transformed beta diversity matrix (Jaccard, Jtu, Jne). Same transformations as S10, 9999 permutations, Metric = component tested, Comparison = pairwise contrast. *F*, pseudo‐*F*; *P*, permutation *p*‐value; *R* ^2^, variance explained. Primer = Combined, Mamm02, V12S‐U. Subset = All_OTUs, named, informative, domestic.
**Table S17:** PERMDISP testing homogeneity of dispersion for each beta diversity component (Jaccard, Jtu, Jne) using betadisper() and permutest() from vegan (9999 permutations). Same matrix transformations as S10. Non‐significant (*p* > 0.05) supports PERMANOVA interpretation. Primer = Combined, Mamm02, V12S‐U. Subset = All_OTUs, named, informative, domestic.
**Table S18:** Cumulative OTU richness at maximum filters (12) per combination from species accumulation curves using specaccum() from vegan (method = random, permutations = 999). richness = mean cumulative richness. SD = standard deviation across permutations. Primer = Combined, Mamm02, V12S‐U. Subset = All_OTUs, Named, Informative, Domestic.

## Data Availability

The collapsed OTU data is available in the Supporting Information to ensure replicable results. Raw sequencing data that support the findings of this study are available on request from the corresponding author. R Code and Data to produce models and figures herein are provided at doi:10.48610/dcedfee. Merged sequencing data, which have been processed for the removal of human DNA sequences, are available at NCBI BioProject PRJNA1506413. We believe the release of the raw files to be in breach of privacy and ethics, given that our data contains a high proportion of DNA that can be assigned to humans, particularly given the sensitivity surrounding Aboriginal and Torres Straight Islander groups.
