# Supplementary material for: A Comparison between Passive airDNA Collection and Extraction Methods on the Recovered Terrestrial Vertebrate Assemblage
Source: Ecol Evol. 2026 Aug 12;16(8):e74135. doi: 10.1002/ece3.74135 (PMC13467694; doi:10.1002/ece3.74135)
Supplement: Supplementary file 1 [file ECE3-16-e74135-s001.zip › ece374135-sup-0003-Supinfo.docx]

Supplementary Material

A comparison of passive airDNA collection and extraction methods on the recovered terrestrial vertebrate assemblage

Jarred Moreno^1^, Nicola Jackson^1^ and Celine Frere^1^

1. School of the Environment, University of Queensland, St Lucia, QLD, Australia.

# Supplementary Methods

## Filter Design

**Sterilisation Procedure**

Sterilisation was achieved by washing the material in a 10% bleach solution, drying it using a KimWipe, washing it again in a 70% ethanol solution, followed by drying it again using a KimWipe and set within a sterile laminar flow cabinet under UV for 20 minutes.

**1. PCR Tape**

2.5” 409-grade stainless steel exhaust pipe (DEA Exhausts) were cut into 33 mm lengths, and a 1mm indent was made at the ‘top’ of each length on both sides of the metal ring, which worked to hold the sterile wire in place. Following this, the 1 mm wire was attached, and the unit was then sterilised. Each unit was sterilised, following which, sterile double-sided tape was added both around the centre of the filter unit, and around the edge of the filter unit. Adhesive PCR plate seals (ThermoFisher) were cut into six equal portions, and four were stuck to each filter unit, with two being placed across the opening of the filter unit and two being placed on each side of the top edge of the filter unit (Figure 1a). The PCR seal storage tab was left on at this stage, and the unit was placed in a sterile autoclave bag and sealed until deployment.

**2. Cloth**

The cloth filters were constructed by first bending a 50 mm BSP Flat Stainless Steel Cover Plate (Plumbing Supplies Australia) at a 45° angle. Sterile 1 mm wire was then tied around each of the bend sites so that the ‘V’ shape was always upright (Figure 1b). Each filter unit, along with two 25 mm bulldog clips, was then sterilised. Following sterilisation, a single, individually packed 7.5 x 7.5 cm Leukoplast sterile compress cotton gauze (Chemist Warehouse) was placed alongside the filter and clips in a sterile autoclave bag and sealed until deployment.

**3. Paraffin Gauze**

The paraffin gauze filters were constructed by drilling a hole into the bottom of a sterile 50 mm falcon tube. A piece of 1 mm wire was then threaded through this hole and screwed into place using the lid (Figure 1c). These were then packed into sterile autoclave bags, each with an individually packaged sterile paraffin gauze, and sealed until deployment.

## Sample Preparation Steps

**1. PCR Tape**

The samples were defrosted by rocking gently on a platform rocker overnight to dislodge particulate matter from the filters. The samples were then vortexed for 30 seconds to ensure that the particulate matter was dislodged. Following this, the samples were swabbed with a sterile FLOQ swab to finally dislodge any remaining particulate matter caught upon the tape. The tip of the swab was broken off using sterile tweezers, the sample was shaken, and then the tape filter was removed from the tubes with sterile tweezers and wiped with sterile tissue paper between each sample. The samples were then centrifuged at 16,639g for 12 minutes, after which the supernatant was removed leaving an approximately 700 µL pellet of DNA.

**2. Cloth**

For DNA extraction, 15 mL of molecular grade PBS (Gibco) was placed directly into 50 mL falcon tubes, so to saturate the gauze filter. Filters were then rocked gently on a platform rocker for 1 hour to dislodge particulate matter from the filters and to defrost the sample. The gauze was then removed from the tubes with sterile tweezers. The samples were then centrifuged at 16,639g for 12 minutes, after which the supernatant was removed leaving an approximately 700 µL pellet of DNA.

**3. Paraffin Gauze**

The initial preparation for the paraffin gauze extraction differed slightly for each extraction kit, however, the initial preparation followed a similar process. To extract the paraffin gauze, 500 µL of sterile PBS was added to the 2 mL collection tube to soak the gauze. The sample was then heated at 56°C for 10 minutes, followed by a 10-second vortex, and centrifuged at 15,000 rpm for 5 minutes.

## Extraction Protocols

**1. Qiagen DNeasy Blood & Tissue Extraction Protocol**

The pellet was resuspended in 180 µl of Buffer ATL, moved to a new 2 mL microcentrifuge tube (excluding paraffin gauze which remained in the initial tube for best lysis potential), and 20 µL of Proteinase K was added, vortexed briefly, and heated to 56°C for 1 hour. Following this, the DNA was purified using a DNeasy Blood & Tissue kit (QIAGEN, Hilden, Germany) following the manufacturer's protocol from step 2. DNA was eluted by passing 2 x 50 µL (a total elution volume of 100 µL) of Buffer AE through the spin column, which was incubated for five minutes on a benchtop at room temperature. Extractions were then stored at -20°C until further analyses.

**2. Qiagen DNeasy PowerSoil Pro Kit**

The pellets from the three preparation methods were transferred into a PowerBead Pro Tube, and 300 µL of solution of CD1 was added. Following this, the DNA was purified using a DNeasy PowerSoil Pro kit (QIAGEN, Hilden, Germany) following the manufacturer's protocol from step 2. Step 2 was completed using the protocol for the TissueLyser II. Throughout this extraction, maximum volumes of solutions and supernatants were taken at each step, i.e. 600 µL of supernatant in step 4, and 700 µL of supernatant in step 6.

**3. MagMAX Nucleic Acid Isolation Kit**

The samples were extracted as per the manufacturer’s protocol for the MagMAX Nucleic Acid Isolation Kit with some slight adjustments. A major adjustment to the protocol was the processing of the samples in 2 mL microcentrifuge tubes using magnetic racks, as I did not have access to the specified Magnetic-Ring Stand (96 well) (Cat. No. AM10050). In addition, at each ‘plate shaking’ step, the samples were briefly vortexed to ensure that the magnetic beads were equally distributed throughout the samples and then shaken as per the manufacturer’s instruction. During the shaking step, all 2 mL microcentrifuge tubes were placed in a way on a 2 mL tube holder to prevent them from freely spinning so that adequate shaking was achieved. The ‘Disruption of liquid samples’ guidelines were used for this extraction using a TissueLyser II. The TissueLyser II guidelines were kept the same as recommended for the PowerSoil Pro extraction kit, with a 5-minute beat at 25 Hz, followed by flipping the samples and beating for another 5 min at 25 Hz. For the final elution, to keep similar to all other extraction kits, 50 µL of elution buffer was processed through the sample twice during step 8, with a 5 minute benchtop incubation for the first elution, resulting in a final elution volume of 100 µL.

**4. Quick-DNA Miniprep Plus Kit (Zymo)**

The extraction was processed as per the manufacturer’s protocol from step 1, with 200 µL of BioFluid & Cell Buffer and 20 µL of Proteinase K added to the sample. For step 2, the incubation was increased to 1 hour to allow for the lysis of sample left within the PCR swab or paraffin gauze. For step 3, one volume of Genomic Binding Buffer was 720 µL to match the current volume in the tube. The rest of the extraction followed the manufacturer’s guideline, with step 8, the final elution, being adjusted to two 50 µL additions of DNA elution buffer, with the 5 min incubation only for the first addition.

## QUBIT Fluorometry

A Qubit 4 Fluorometer was used to measure the concentration of DNA within each sample, to understand if any extraction kit or filter type differed in the concentration of DNA collected. This was completed as per the manufacturer’s instructions with 20 µL of sample used for each reaction.

# Supplementary Figures

## Supplementary Figure 1:

Supplementary Figure 1: Dotplot showing the relationship between the total number of unique ASVs recorded on each sample and the QUBIT concentration for each sample, with the unique combination of filter type (circle = cloth, triangle = paraffin) and extraction kit (orange = Blood and Tissue, green = MagMAX, blue = PowerSoil or purple = Zymo) outlined.
